# Cardiac well-being indexes: a decision support tool to monitor cardiovascular health

**DOI:** 10.1515/jib-2020-0040

**Published:** 2021-03-29

**Authors:** Ana Duarte, Orlando Belo

**Affiliations:** Algoritmi R&D Centre, University of Minho, Campus of Gualtar, 4710-057, Braga, Portugal,

**Keywords:** analytical systems, data mining, decision support systems, heart disease prevention, well-being indexes

## Abstract

Despite the increasing awareness about its severity and the importance of adopting preventive habits, cardiovascular disease remains the leading cause of death worldwide. Most people already recognize that a healthy lifestyle, which includes a balanced diet and the practice of physical activity, is essential to prevent this disease. However, since few simple mechanisms allow a self-assessment and a continuous monitoring of the level of cardiac well-being, people are not conscious enough about their own cardiovascular health status. In this context, this paper presents and describes a tool related to the creation of cardiac well-being indexes that allow a quick and intuitive monitoring and visualization of the users’ cardiovascular health level over time. For its implementation, data mining techniques were used to calculate the indexes, and a data warehouse was built to archive the data and to support the construction of dashboards for presenting the results.

## 1 Introduction

In today’s society, especially in more developed countries, *cardiovascular disease* (CVD) is of particular importance since it has a high mortality rate but, at the same time, many of its risk factors can be prevented, either by medication or by a healthier lifestyle [1]. However, with the fast pace of modern life and the resulting lack of time, health is often overlooked, and healthy lifestyle habits neglected.

To monitor and control some clinical parameters, people generally do regular clinical analysis. Any of those parameters, such as the levels of LDL cholesterol, being outside of the normal range will certainly raise special care. People will, probably, correct certain behaviours in order to normalize those parameters. Thus, similarly to clinical analysis, it would be useful to have a simple mechanism to assess general cardiovascular health without requiring specialized medical expertise. Similarly, a low value indicating a poor cardiovascular health would be enough to make people rethink their habits and to encourage a change in their behaviour, acting as a warning in order to maintain the cardiovascular well-being.

One way to permit this analysis is through the use of informatics tools that enable cardiovascular health to be quantified and presented in an easily understandable form. These tools allow the identification of the main risk factors and their correlation with the development of CVD, and they also offer significant advantages in terms of communication and dissemination of knowledge about the disease. One example is the possibility of generating and making available intuitive cardiovascular graphs both to the population and to health professionals.

In this way, we implemented a decision support tool for the creation of cardiac well-being indexes to promote cardiovascular health and reduce the incidence of this type of disease. In the next section of the paper, we briefly discuss the Framingham Heart Study and some limitations of the existent cardiac risk simulators. Sections 3–5 describe, respectively, the method of calculating the indexes, the analytical system we built to store all records, and the results we obtained. Finally, in Section 6 we summarize the main conclusions and some possible future work.

## 2 Related works

Before the 1940s, cardiovascular problems were thought to be inevitable and a consequence of the normal ageing process. During this decade, CVD was the main cause of death in the USA and, even, American President Franklin Roosevelt died victim of the disease. These factors, combined with the poor understanding of cardiovascular disease, triggered, in 1948, the development of the *framingham heart study* (FHS), in Massachusetts, USA. The FHS is the longest and most important epidemiological study on CVD. It began with a total of 5209 participants living in the city and aged between 28 and 62 years. The average age of the participants was 44 years, and more than half were female [1], [2].

As a result of the study, in 1961, one of the most important reports was published, in which gender, age, blood pressure, cholesterol and left ventricular hypertrophy were identified as risk factors associated with CVD [3]. Subsequent studies conducted between 1962 and 1964, demonstrated the existence of a direct relationship between being a smoker and the increased risk of CVD. In 1967, it was found that physical activity was inversely proportional to the degree of risk of developing the disease. In the following years, obesity, atrial fibrillation and diabetes mellitus were also included in the list of risk factors [2].

Based on the results of the FHS and the scores attributed to the main risk factors, a methodology was published in 1998 that enable the prediction of the likelihood of contracting CVD at ten years. According to this mechanism, the scores associated with each risk factor are summed, and the result corresponds to a tabulated risk percentage based on the total of points [4].

A direct consequence of the establishment of this methodology and other studies developed in the field of CVD risk factors is the possibility of implementing calculators which allow the estimation of cardiac risk. Several tools are currently available for this purpose, such as [5], [6], which allow users to calculate their own cardiac risk. However, a downside of these calculators is the fact that they return a percentage that is not easy to understand by common users. For example, a risk value of 10% does not give a clear perception to understand whether that value corresponds to a high or low risk. Adjusting the range scale to more intuitive values could be a solution to overcome this limitation. Moreover, the existent calculators use a methodology that considers only a few risk factors, since they cannot handle complex relationships between the various attributes. In this sense, *data mining* (DM) techniques make it possible to overcome this limitation by using models with many features and multiple correlations between them.

Regarding the use of these techniques to predict the risk of CVD, there are already several studies that have demonstrated their effectiveness, such as (7). In this study, the authors used decision trees, neural network, Bayesian model and support vector machine models to predict heart attacks and to verify whether the incidence of CVD was higher in coal mining regions than in other locations.

In another study, conducted by Karaolis et al. [8], DM techniques were used to determine the main risk factors associated with myocardial infarction events, percutaneous coronary intervention and coronary artery bypass surgery. After performing the DM process, the results showed that age, smoking and hypertension were the main risk factors for myocardial infarction and coronary artery bypass surgery. In its turn, regarding percutaneous coronary intervention, the main risk factors found were family history, hypertension and diabetes.

In a similar study, authors Kim and Kang used neural networks to highlight the main risk factors associated with coronary heart disease. The results demonstrated that the most significant attributes were age, *body mass index* (BMI), total cholesterol, HDL cholesterol, systolic blood pressure, diastolic blood pressure, triglycerides and diabetes [9].

Apart from those shortcomings, existent calculators are more indicated for sporadic measurements, as they only consider the current values. Thus, all previous measurements are ignored, and the result is more prone to errors due to the possibility of existing outliers. In this way, these calculators are not suitable for continuous monitoring, since they do not store the history of each user’s measurements and do not take into account the variations of the risk over time. Such monitoring would make it possible to assess the evolution of the risk more accurately and would also detect the presence of atypical measurements, thereby mitigating their influence on the calculation of the risk.

One possible approach to solve this is to store the data in a *data warehouse* (DW). The implementation of this type of repository permits to archive all records over time, in a well-organized and consistent manner. Furthermore, data warehouses also enable the integration with other tools, such as dashboards [10].

Dashboards allow the results to be presented in a simple, interactive and easily understandable way. Their advantages in the medical field have been highlighted in several studies, such as [11], in which the authors created dashboards to visualize the stream of clinical data in real-time.

There are many different possible methods for creating cardiac well-being indexes. However, the integration of DM techniques, *data warehousing systems* (DWS) and dashboards seems to be a promising approach to establish reliable and intuitive indexes, capable of encouraging the monitoring of cardiovascular health and, thus, promoting cardiac well-being.

## 3 Building a predictive model

Cardiac well-being indexes can be calculated using DM techniques, in order to consider many risk factors and the many interactions between them. To support this step, we used a dataset adapted from Kaggle [12], containing classified data regarding the existence of CVD. In addition to the classification attribute, the dataset comprised a total of 17 attributes related to the CVD risk and 65,000 records. Tables 1 and 2 present, respectively, the general statistics for each numerical and nominal attribute of the dataset.

**Table 1:** General statistics of numerical attributes used for the data mining process (before the process of data cleansing).

Numeric attribute	Min	Max	Mean	Standard deviation
Age	29	64	52.8	6.8
Systolic blood pressure	−150	16020	128.9	159.6
Diastolic blood pressure	−70	11000	96.9	188.1
Cholesterol	100	320	170.1	52.5
Fast glucose	80	400	119.6	56.4
Smoking years	0	50	5.4	12.0
Number of cigarettes	0	50	7.1	14.6
BMI	3.5	298.7	27.6	6.1

**Table 2:** General statistics of nominal attributes used for the data mining process (before the process of data cleansing).

Nominal attribute	Missing values	Value	Occurrences
Family history	0	Yes	8,459
		No	56,541
Pain after effort	0	Yes	8,620
		No	56,380
Physical exercise	0	None	6,352
		Low	6,413
		Moderate	26,054
		High	26,181
Smoking	34,956	Yes	6,651
		No	23,393
Hypertension	0	Yes	25,225
		No	39,775
Hypothyroid	0	Yes	13,501
		No	51,499
Gender	0	Male	28,326
		Female	36,674
Record date	0	—	—
Diabetes	65,000	—	—
Class	0	Disease	32,509
		No disease	32,491

In the first stage, *pentaho data integration* (PDI) was used to cleanse the data. First, the data were extracted from the source, and all fields were sorted. Then, the records were filtered, and outdated instances and records containing inconsistencies or incoherencies were excluded. The data can be automatically prepared and transformed in PDI each time a DM process is executed. As a result, this process can be performed in an automated way, without requiring human intervention. However, to ensure its feasibility, periodic maintenance is also needed.

After the pre-processing phase, the treated data were saved in the form of an “arff” file to enable the automatic communication between PDI and Weka, using the knowledge flow plugin [13], [14]. Weka was the software used for the train and the test of the DM models.

In DM processes, reducing the number of attributes under analysis can bring some benefits, such as a significant reduction in the execution time of the algorithms or even improvements in terms of predictive capacity, by eliminating less relevant attributes or attributes with a certain degree of interdependence. For this reason, in order to consider the set of attributes associated with the best results, two scenarios were defined:

1. Scenario I: containing all the 17 risk factors
2. Scenario II: containing only the most relevant risk factors filtered by Weka’s attribute selection

The DM techniques selected to build the models were J48, random forest (RF), naïve Bayes (NB), k-nearest neighbors (KNN) and MultiLayerPerceptron (MLP), since they have already demonstrated their efficiency in similar studies [15–18]. To optimize the parameters for each technique, we performed a total of 870 different simulations using 10-fold cross-validation. According to [19], 10 is the standard number of folds to use in cross-validation strategies and provides, in general, the best results. Table 3 summarizes the best parameters found for each technique. After replacing the default values of the parameters of each technique by the optimized values, the models were generated.

**Table 3:** Optimized parameters for each data mining technique.

J48	RF	NB	KNN	MLP
ConfidFactor: 0.10	NumTrees: 80	KernelE: F	KNN: 20	HiddenLayer: a
MinNumObj: 20	MaxDepth: 25	SupervD: T	CrossValid: F	TrainTime: 100
RedErrorPrun: T	NumFeat: 0		DistWeight: No	NominalToBin: T
Unpruned: F	BreakTies: T		SearchA: KDTree	Decay: F
NumFolds:				Lr: 0.1
Scenario I: 6				Momentum: 0.05
Scenario II: 3				
MDLcorrection: T				

The chosen model must not only present correct values, but it should also properly identify most sick people, even if in some cases this implies an incorrect classification of healthy people. Therefore, for the selection of the best algorithm, the metrics considered were accuracy and sensitivity. Accuracy is directly related to the ability to recognise sick and healthy people correctly. On the other hand, sensitivity measures the effectiveness in identifying individuals with disease in the universe of patients.

In order to establish an automatic mechanism for selecting the best model, PDI was used. It was assumed that accuracy and sensitivity have the same relevance in the prediction of CVD. Thus, we considered that the best model is the one that sums the highest scores considering the average of both metrics. The results obtained, presented in Table 4, allowed an orderly ranking of the best models for predicting CVD: MLP (0.7265) > RF (0.7255) > J48 (0.7225) > ASJ48 (0.719) > ASKNN (0.7180) > ASMLP (0.7150) > ASRF (0.7115) > KNN (0.7065) > NB (0.6930) > ASNB (0.6865). Note that the prefixes “AS” refer to the models created using the features from scenario II.

**Table 4:** Sensitivity, accuracy and score values obtained for training data.

Algorithm	Sensitivity	Accuracy	Score
MLP	0.700	0.753	0.7265
RF	0.703	0.748	0.7255
J48	0.694	0.751	0.7225
ASJ48	0.688	0.750	0.7190
ASKNN	0.701	0.735	0.7180
ASMLP	0.684	0.746	0.7150
ASRF	0.692	0.731	0.7115
KNN	0.685	0.728	0.7065
NB	0.647	0.739	0.6930
ASNB	0.638	0.735	0.6865

To validate the results obtained, we used a new dataset containing 5,000 records with the same attributes. The sensitivity and accuracy values found for this dataset are indicated in Table 5. Since these values remained identical to those obtained in the training dataset, the results were considered valid.

**Table 5:** Sensitivity and accuracy values obtained for testing data.

Algorithm	Sensitivity	Accuracy
MLP	0.7117	0.7232
RF	0.7093	0.7288
J48	0.6895	0.7192
ASJ48	0.7130	0.7294
ASKNN	0.7121	0.7074
ASMLP	0.6980	0.7372
ASRF	0.7024	0.7206
KNN	0.6931	0.7032
NB	0.6595	0.7418
ASNB	0.6445	0.7380

Afterwards, we established that the indexes vary between −5 and 5. In this way, the negative values can be immediately associated with a poor cardiovascular health, and the positive values with a satisfactory cardiovascular health. Each index value also corresponds to one of the following colours: green (for values between 2.5 and 5), yellow (for values between 0 and 2.5), orange (for values between −2.5 and 0), or red (for values between −5 and −2.5). The values of the indexes are determined by converting the probabilistic values associated with the classes “disease” and “no disease”, obtained using MLP algorithm (scenario I), into a value between −5 and 5. However, this conversion must not be done linear, as the indexes should be able to detect high risks of developing CVD at an early stage. It is preferable to incorrectly alert a healthy person to a false risk of developing CVD than to classify as healthy someone who is at high risk of developing the disease. Therefore, the indexes must decrease exponentially towards an increase in the risk of developing the disease. For this reason, we constructed an exponential curve considering a function *f*(*x*) = *A* + *B* exp(*Cx*). The curve is represented by Eq. (1), assuming that:

– A probability of 0% of having a CVD corresponds to the index value 5.
– A probability of 100% of having a CVD corresponds to the index value −5.
– A probability of 55% of having a CVD corresponds to the threshold between the colours orange and red (index value −2.5).

(1) $$f\left(x\right)=-7.052755+12.05276\times e^{-1.77011x}$$

Applying the developed mechanism to the data under study, it was found that most people with CVD had a corresponding red index, and less than 2% of them had a green index. These facts proved the appropriateness of the proposed methodology and the use of MLP as the algorithm for calculating cardiac well-being indexes.

Having already established a methodology for calculating the indexes, it is now possible to quantify the cardiovascular well-being. However, it is still necessary to take past measurements into account in order to reflect the entire history.

## 4 Storing the indexes

**Figure 1:**
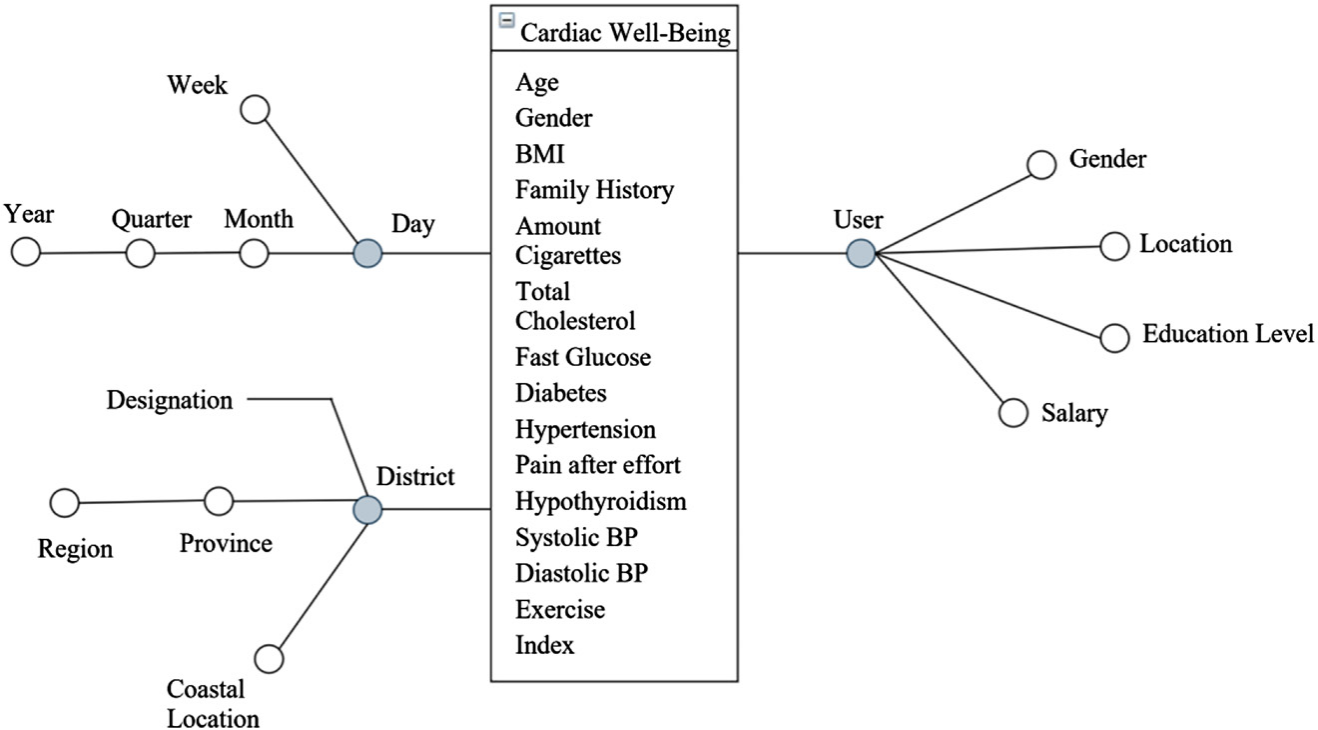
The multidimensional schema of the data warehouse.

The constructed DM model and Eq. (1) can be used as a mechanism to calculate the indexes of the users. If users take multiple measurements over time, a repository such as a DW enables the achievement of historical records in a consistent and coherent manner. With the developed calculation mechanism, it is possible to store the indexes in a DW and to create weighted indexes, considering not only the current measurements but also the past values. As a result, the data in the DW can be used for general statistics about the cardiovascular health of the population or even to monitor and analyse the evolution of the index of a particular user. Thus, after implementing a calculation mechanism, we conceived a DW to archive the indexes following the Kimball 4-step methodology [10]. The DW was designed according to the conceptual schema illustrated in Figure 1.

In this process, we used three new data sources containing users’ records and geographical information about the districts of Portugal:

– Source 1: personal/clinical data from users that do not vary frequently (or do not vary at all)
– Source 2: personal/clinical data from users that vary frequently (e.g., daily calories spent)
– Source 3: data containing information about the administrative regions of Portugal

Both sources 1 and 2 were created using synthesized data and simulate possible records of users who intend to calculate their cardiovascular well-being, whereas source 3 was created based on real data. Initially, to assess the quality of the data, we performed a data profiling task using SQL server data tools from visual studio, through the data profiling task editor. This analysis provides some useful information about null and unique values, the distribution of the variables, and the data format and size [20]. The results of the data profiling task demonstrated, in general terms, that the required data were available and that the data were of high quality.

**Figure 2:**
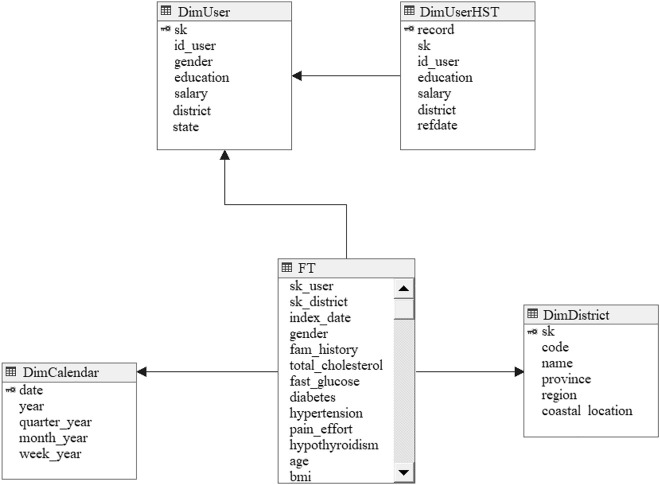
Implemented structures in the data warehouse.

The physical schema of the DW follows the structure depicted in Figure 2. In addition to the fact table and the dimensions *DimUser*, *DimCalendar* and *DimDistrict*, the dimension table *DimUserHST* was also created to archive all the detected changes in the values of the users’ attributes with a temporal indication.

Since not all data have a direct correspondence between the source and the destination, we structured the source to target mapping represented in Table 6. This source to target mapping describes the necessary transformations to migrate the data from the different sources to their corresponding structure in the DW.

**Table 6:** Source to target mapping. Data from the various sources (left) and their transformations before being loaded into the target (right), in the Data Warehouse^a^.

Source	Attribute		Transformation		Attribute	Target
Source 1	Id	Int	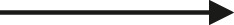	Int	Id_user	Dim user
	District	Varchar	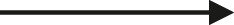	Varchar	District	
	Education level	Varchar	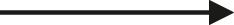	Varchar	Education	
	Salary	Int		Varchar	Salary	
	Gender	Varchar	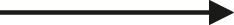	Varchar	Gender	
		Varchar	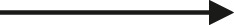	Varchar	Gender	
	Weight	Int	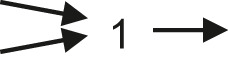	Float	bmi	Fact table
	Height	Int				
	Family history	Varchar	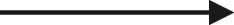	Varchar	Fam_history	
	Total cholesterol	Int	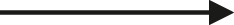	Int	Total_cholesterol	
	Fast glucose	Int	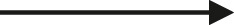	Int	Fast glucose	
	Diabetes	Varchar	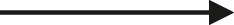	Varchar	Diabetes	
	Hypertension	Varchar	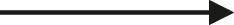	Varchar	Hypertension	
	Pain after effort	Varchar	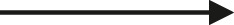	Varchar	Pain_effort	
	Hypothyroidism	Varchar	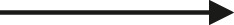	Varchar	Hypothyroidism	
	Clin. Analysis date	Date	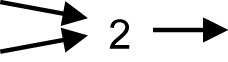	Int	Age	
	Date of birth	Date				
	Numb. Cigarettes	Int	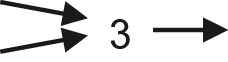	Float	Smoke_amount	
	Smoke years	Int				
Source 2	Id	Int	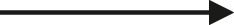	Int	Id_user	Fact table
	Index date	Date	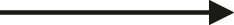	Date	Index_date	
	Calories	Int	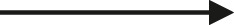	Varchar	Physical_exercise	
	Systolic BP	Int	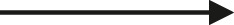	Int	Systolic BP	
	Diastolic BP	Int	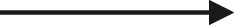	Int	Diastolic BP	
Source 3	Code	Varchar	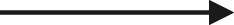	Varchar	Code	Dim district
	Name	Varchar	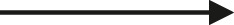	Varchar	Name	
	Province	Varchar	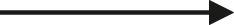	Varchar	Province	
	Region	Varchar	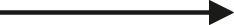	Varchar	Region	
	Coastal location	Varchar	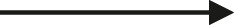	Varchar	Coastal location	

^a^1 – $BMI=\frac{wei\text{g}ht}{{hei\text{g}ht}^{2}}$ , 2 – age=year(clinicalanalysisdate−dateofbirth) , 3 – $smoke\;amount=\frac{365\times number\;ci\text{g}arettes\times smokin\text{g}\;years}{1000}$

**Figure 3:**
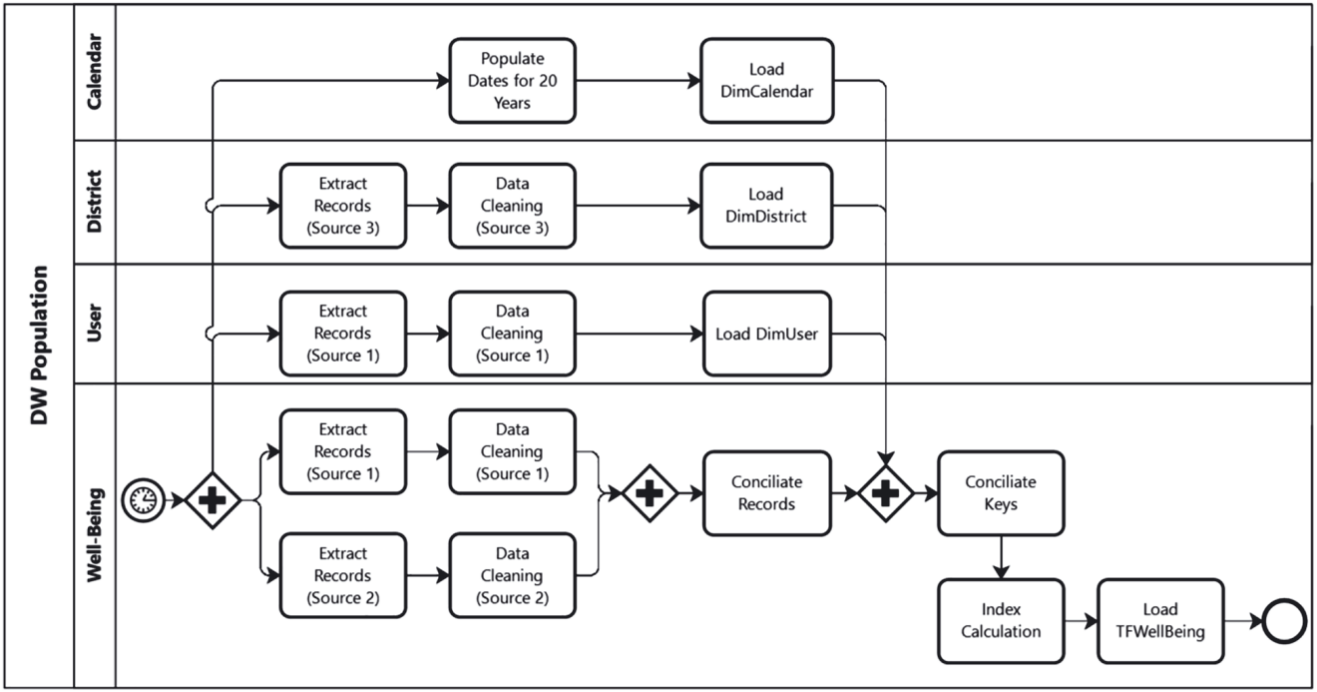
Methodology used to populate the tables of the data warehouse (BPMN schema).

The principal tasks of the *extraction, transformation and loading* (ETL) process are represented in Figure 3, using *business process modelling notation* (BPMN). It is necessary to populate the dimension tables first, and only then the fact table can be loaded into the DW. Since the data in *DimCalendar* and *DimDistrict* cannot be modified, these dimension tables are static, and their data are loaded only once in a full loading process. Conversely, *DimUser* and the fact table require incremental loading processes, since these tables are populated with dynamic data that are subject to change. In the case of the fact table, a mechanism to capture only the records loaded after the execution of the previous ETL was created. On the other hand, the data capture mechanism considered for *DimUser* consisted in the use of triggers to enable the capture of new, modified and removed records. This dimension was considered a *slowly changing dimension* (SCD) type 4 to enable the achievement of users’ historical data in the *DimUserHST* [21].

The ETL was programmed to run daily and the DM process monthly. Both tasks can be started and executed automatically. The DM job selects and stores in a file the name of the best algorithm to support the calculation of the indexes. On the other hand, when the ETL job is executed, the new and changed data are extracted from the sources, transformed, and loaded into the corresponding tables. The indexes are loaded into the DW at the end of the “index calculation” step (Figure 3). During the execution of this step, the DM model with the highest score is used as the basis for the calculation of the indexes.

Finally, the indexes must be arranged according to the different visualization perspectives. For this reason, the creation of an on-line analytical processing (OLAP) cube enabled the construction of individual and global indexes. The individual indexes result from weighting the historical values according to their evolution over time. In this way, outliers are minimized and the value presented to the user already reflects all his/her previous measurements. For this purpose, the *LinRegPoint* function was used to calculate the linear regression line and return the value of the weighted index for a given date. On the contrary, the global indexes are calculated taking into account all unweighted indexes of all users.

## 5 Creating cardiac well-being dashboards

To visualize the indexes from multiple perspectives, we created dashboards in three different pages, using Power BI (22). These pages were organized as follows:

1. First page: dashboards for individual users and health professionals
2. Second page: dashboards for statistical analysis
3. Third page: users’ history table for further analysis

Thus, the first page created enables the monitoring of the weighted indexes over time. This dashboard can be a useful tool, not only for the individual users to have a regular control on their cardiovascular health, but also for health professionals to assist their patients. To provide a more intuitive analysis, each value was associated with its corresponding colour – green, yellow, orange or red. This dashboard allows users to navigate through the calendar hierarchy to view the indexes by year or month, for example.

**Figure 4:**
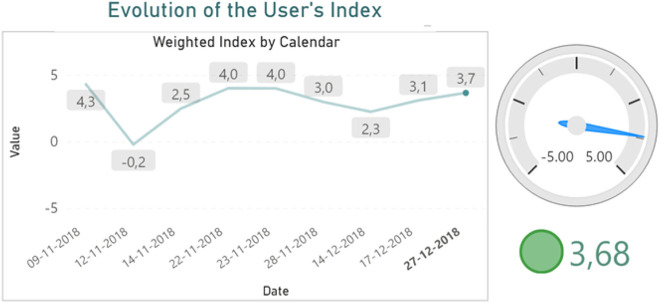
Dashboards to evaluate the cardiac well-being indexes of a user over time.

Figure 4 presents the results obtained for the user with ID 2961. In this case, it can be observed that the user’s current index is 3.68, which consequently corresponds to the colour green. The value returned already considers all previous records. In this way, doing this analysis, an individual user can have a better sense of the level of his/her cardiovascular well-being.

In addition to the first type of dashboards, the second page provides a set of indicators mainly aimed at decision-makers in the health sector. These interactive indicators provide global information, that is particularly useful for statistical analysis, and a broader perception of the level of cardiovascular health in the population.

**Figure 5:**
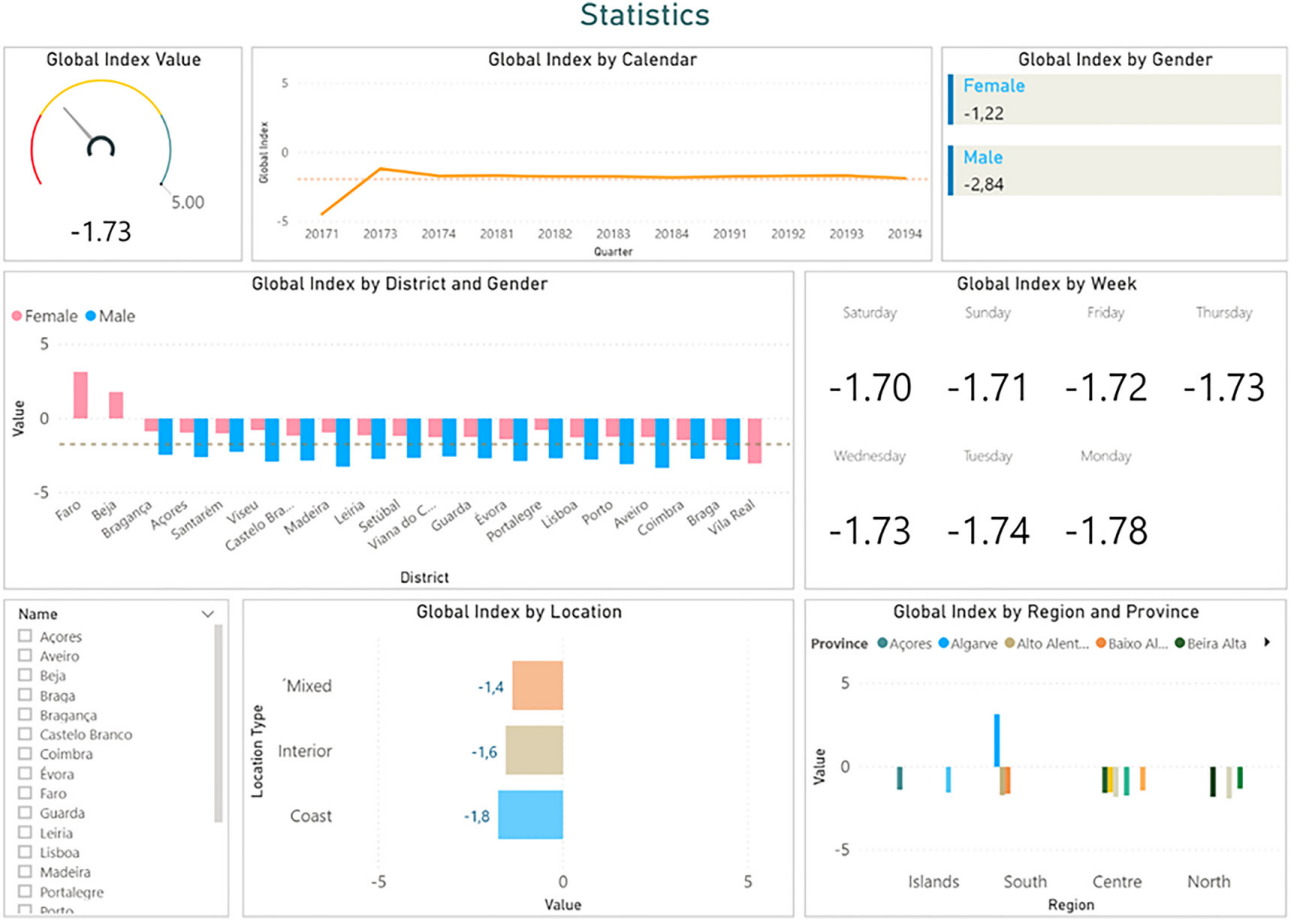
Dashboards regarding global cardiac well-being indexes for statistical analysis.

In these dashboards, the main indicator is the global value of the index. In the case of the example in Figure 5, this value is −1.73. Since it is negative, it indicates that the cardiovascular health of the population is generally not properly maintained. For this reason, awareness campaigns could be launched to improve the global index.

Similar to the dashboards on the first page, in this case, the global index can also be visualized according to different time periods, such as a specific year, month or day.

In summary, from Figure 5 we can observe that:

– Although there are no significant differences between the days of the week, the worst values are recorded on Mondays.
– According to the gender, although women register better values, both genders present negative indexes.
– Only the Algarve, in the southern region, has positive indexes.

This type of information can be useful to enable health decision-makers to act locally according to the specific needs of each population. In case a more detailed analysis is needed, users can select a specific parameter and, consequently, all indicators will be updated according to the chosen perspective.

Despite the relevance of these dashboards, it must also be possible to access the old users’ records, which are no longer in force. For example, if users change their educational level, it may be important, in the future, to have access to the various updates of this parameter to see whether these changes have affected the values of their indexes.

**Figure 6:**
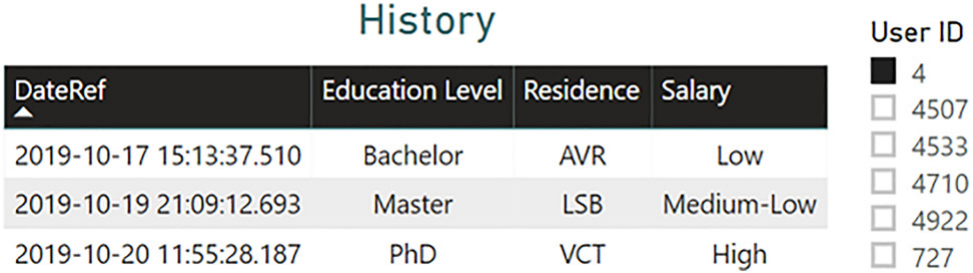
History table of the user with ID 4 containing the modified records regarding education level, residence and salary.

Thus, the third page allows experts to consult the history of users who have changed their records in terms of educational level, residence, and salary. Figure 6 provides an example of the history table created for the user with ID 4.

The table in Figure 6 shows that on 20th October 2019, the user’s residence changed from LSB to VCT. This information could justify cases where the indexes fluctuate significantly.

## 6 Conclusions and future work

The investment in the prevention of cardiovascular problems should include as many resources as possible to significantly reduce the number of deaths caused by CVD. In this context, the key contribution of this work is the development of a tool based on the establishment of cardiac well-being indexes. These indexes are suitable for the continuous monitoring of cardiovascular health, as the results also take into account past values. For this reason, the reliability of the proposed tool increases with the number of measurements performed.

The integration of DM techniques, DWS and dashboards enabled the creation of an effective cardiovascular health alert system. The indexes returned are easy to understand for most people and can therefore contribute to the promotion of cardiovascular well-being. Furthermore, they can also be an aid to assist health professionals or even for health decision-makers. The possibility of visualizing global indicators by specific characteristics, such as gender or location, can help health decision-makers to collect data on the cardiovascular health of the population from different perspectives.

Due to the lack of real data, it was not possible to conduct a more in-depth analysis of the global cardiovascular health in Portugal. Therefore, it would be important for future work to validate the developed tool with real data. Furthermore, security issues were not considered, and the system was not tested in a real context. These situations could also be taken into account for further work.
